# Novel Causal Evidence for the Role of Circulating Lipid Metabolites in Breast Cancer Risk: A Bidirectional Mendelian Randomization Analysis

**DOI:** 10.1155/jnme/2292774

**Published:** 2025-09-16

**Authors:** Cheng Zeng, Shuning Liu, Yuhan Wei, Yalong Qi, Yujing Tan, Haili Qian, Jiani Wang, Fei Ma

**Affiliations:** ^1^Department of Medical Oncology, National Cancer Center/National Clinical Research Center for Cancer/Cancer Hospital, Chinese Academy of Medical Sciences and Peking Union Medical College, Beijing 100021, China; ^2^Department of Clinical Research, Sun Yat-Sen University Cancer Center, State Key Laboratory of Oncology in South China, Guangdong Provincial Clinical Research Center for Cancer, Guangzhou 510060, China; ^3^Department of Oncology, Beijing Friendship Hospital, Capital Medical University, Beijing 100050, China; ^4^State Key Laboratory of Molecular Oncology, National Cancer Center/National Clinical Research Center for Cancer/Cancer Hospital, Chinese Academy of Medical Sciences and Peking Union Medical College, Beijing 100021, China

**Keywords:** causal inference, estrogen receptor-positive breast cancer, lipid metabolites, Mendelian randomization, metabolic biomarkers

## Abstract

**Background:** Dysregulated lipid metabolism has been implicated in breast cancer pathogenesis; however, the causal nature of these associations remains unclear. This study aimed to investigate the causal relationships between circulating lipid metabolites and the risk of estrogen receptor–positive (ER+) and ER-negative (ER−) breast cancer using a bidirectional Mendelian randomization (MR) approach.

**Methods:** We evaluated 386 circulating lipid metabolites as exposures in two-sample MR analyses, with ER+ and ER− breast cancer as outcomes. Genetic instruments were selected based on genome-wide significance (*p* < 1 × 10^−5^) and linkage disequilibrium clumping (*R*^2^ < 0.01 within a 1000 kb window). The inverse variance weighted method was used as the primary analytical approach. Sensitivity analyses—including MR-Egger regression, Cochran's *Q* test, and leave-one-out analyses—were conducted to assess pleiotropy and heterogeneity. Validation analyses were performed using an independent genome-wide association study (GWAS) dataset for ER+ breast cancer. Reverse MR analysis was also conducted to evaluate potential reverse causality.

**Results:** Among the 386 lipid metabolites analyzed, 24 and 23 exhibited nominal associations (*p* < 0.05) with ER+ and ER− breast cancer, respectively. After applying false discovery rate (FDR) correction (FDR < 0.05), three metabolites—myristoleate (14:1n5), tricosanoyl sphingomyelin (d18:1/23:0), and 5α-androstan-3β, 17β-diol monosulfate (2)—remained significantly associated with an increased risk of ER+ breast cancer. In contrast, none of the associations with ER− breast cancer remained significant after FDR correction. Sensitivity analyses indicated no evidence of horizontal pleiotropy or heterogeneity, and the associations remained robust in leave-one-out analyses. These findings were further validated in an independent GWAS dataset. Moreover, reverse MR analysis found no evidence supporting a causal effect of ER+ breast cancer on the levels of the three identified lipid metabolites.

**Conclusions:** This study provides robust genetic evidence supporting a causal role for specific lipid metabolites in the development of ER+ breast cancer. These metabolites may serve as potential biomarkers for early detection and targets for preventive interventions.

## 1. Introduction

Breast cancer is the second most commonly diagnosed malignancy among women worldwide and remains one of the leading causes of cancer-related mortality [1]. As a highly heterogeneous disease, breast cancer is classified into distinct molecular subtypes, among which estrogen receptor-positive (ER+) and estrogen receptor-negative (ER−) breast cancers represent two major clinical categories [2]. These subtypes differ significantly in terms of prognosis, therapeutic response, and underlying pathogenesis [3, 4]. Although considerable progress has been made in identifying genetic and environmental risk factors, the etiology of breast cancer subtypes remains incompletely understood.

Metabolic reprogramming is a hallmark of cancer and plays a pivotal role in tumor initiation and progression [5]. Among the various metabolic pathways, lipid metabolism has attracted increasing attention in hormone receptor–driven malignancies, particularly ER+ breast cancer [6]. Lipid metabolites, as end products of complex enzymatic and genetic regulation, not only reflect systemic metabolic disturbances but may also provide functional insights into cancer development [7–10]. However, the causal relationships between lipid metabolites and specific breast cancer subtypes—especially ER+ and ER− breast cancer—have not been systematically evaluated. Existing studies have largely focused on conventional lipid profiles or a limited number of metabolites, without comprehensive coverage of key small molecules involved in lipid metabolic pathways [11, 12]. Furthermore, the metabolic distinctions between ER+ and ER− subtypes remain underexplored. Observational studies are often subject to confounding and reverse causation, limiting the interpretability and consistency of their findings.

Mendelian randomization (MR) is an analytical approach that leverages genetic variants as instrumental variables (IVs) to infer causal relationships between exposures and disease outcomes [13]. By mimicking the randomization process of clinical trials, MR can effectively minimize the influence of confounding factors and reverse causality [14]. In recent years, MR has been widely adopted in cancer epidemiology to uncover etiological mechanisms and prioritize modifiable risk factors for prevention and intervention [15]. Although several MR studies have explored associations between metabolites and breast cancer risk, most have examined general metabolic features or common lipids and have been limited in scope regarding lipid metabolite coverage [11, 12, 16, 17]. Additionally, inadequate control for multiple testing has compromised the statistical robustness of some findings. To date, few studies have systematically investigated the causal roles of circulating lipid metabolites in ER+ versus ER− breast cancer, leaving the metabolic heterogeneity across subtypes largely unresolved.

To address these gaps, we conducted a two-stage MR study to comprehensively assess the causal effects of 386 circulating lipid metabolites on ER+ and ER− breast cancer. In the first stage, we identified three lipid metabolites—myristoleate (14:1n5), tricosanoyl sphingomyelin (d18:1/23:0), and 5α-androstan-3β,17β-diol monosulfate (2)—that exhibited significant causal associations with ER+ breast cancer after false discovery rate (FDR) correction. These associations were further validated using an independent genome-wide association study (GWAS) dataset. In contrast, no lipid metabolites were found to be causally associated with ER− breast cancer. In the second stage, reverse MR analysis provided no evidence that ER+ breast cancer influences the levels of these metabolites, supporting a unidirectional causal relationship. Collectively, our findings highlight the potential etiological relevance of lipid metabolism in ER+ breast cancer and suggest that specific circulating lipid metabolites may serve as early biomarkers or therapeutic targets for this subtype.

## 2. Materials and Methods

### 2.1. Study Design

**Figure 1:**
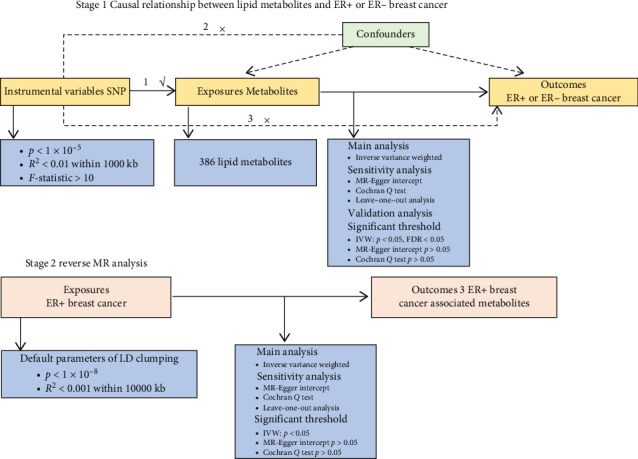
Overview of the two-stage MR analysis. In Stage 1, MR was performed to assess the causal effects of 386 lipid metabolites on ER+ and ER− breast cancer using IVW as the primary method, with multiple sensitivity and validation analyses. In Stage 2, reverse MR analysis was conducted to evaluate whether ER+ breast cancer causally influences the three identified lipid metabolites. The MR analysis relied on three assumptions: (1) SNPs must be associated with exposure, (2) SNPs must not be associated with any confounding factors, and (3) SNPs must not be directly associated with any outcome. ER, estrogen receptor; MR, Mendelian randomization; SNPs, single-nucleotide polymorphisms; FDR, false discovery rate; IVW, inverse variance weighted.

This study employed a bidirectional MR framework to investigate the causal relationships between circulating lipid metabolites and the risk of breast cancer stratified by ER status, as outlined in Figure 1. In Stage 1, forward MR analyses were conducted to assess the causal effects of 386 lipid metabolites on ER+ and ER− breast cancer. In Stage 2, reverse MR analyses evaluated whether ER+ breast cancer causally influenced the levels of the identified lipid metabolites.

MR analyses were conducted under three core assumptions: (1) genetic variants (single nucleotide polymorphisms, SNPs) are strongly associated with the exposure; (2) SNPs are not associated with confounding variables; and (3) SNPs influence the outcome only through the exposure. The latter two assumptions were evaluated through a series of sensitivity analyses. All summary-level data were obtained from publicly available GWAS, each of which had received appropriate ethical approval and informed consent from participants. This study adhered to the STROBE-MR reporting guidelines [15].

### 2.2. GWAS Data Sources for Exposure and Outcomes

GWAS summary statistics for 386 circulating lipid metabolites were obtained from a large-scale metabolomics GWAS conducted by Chen et al. [18], primarily involving individuals of European ancestry (Table S1). In this study, plasma concentrations of lipid metabolites were quantified using ultrahigh-performance liquid chromatography coupled with tandem mass spectrometry (UPLC–MS/MS), utilizing the Metabolon HD4 platform. To ensure robust and accurate metabolite profiling, stringent quality control and data curation protocols were applied. Metabolites with greater than 50% missingness were excluded, and the remaining values were batch-normalized to account for systematic variation. Subsequently, data were log-transformed, outliers beyond three standard deviations were removed, and values were standardized using z-transformation (mean = 0, standard deviation = 1). GWAS analyses were conducted using linear regression models implemented in the fastGWA tool (GCTA v1.93.2beta) [19], adjusting for key covariates including age, sex, fasting status, genotyping batch, and the first ten genetic principal components. These preprocessing and analysis steps ensured the reliability and reproducibility of the metabolite–SNP association estimates. Additional methodological details can be found in the original publication by Chen et al. [18].

Outcome data for ER+ and ER− breast cancer were retrieved from the IEU OpenGWAS project (https://gwas.mrcieu.ac.uk/), all based on European populations and aligned to the HG19/GRCh37 genome build. The ER+ breast cancer dataset (ieu-a-1127), derived from the Breast Cancer Association Consortium (BCAC) and published by Michailidou et al. [20], included 175,475 female participants, with 69,501 ER+ breast cancer cases and 105,974 controls, encompassing 10,680,257 SNPs. The ER− breast cancer dataset (ieu-a-1128) consisted of 127,442 individuals, including 21,468 ER− breast cancer cases and the same control group of 105,974 individuals, with equivalent SNP coverage. To validate the findings from the primary MR analysis, an independent dataset for ER+ breast cancer (ieu-a-1132) was used. This replication cohort included 83,691 participants (38,197 ER+ breast cancer cases and 45,494 controls) and covered 10,680,257 SNPs. All datasets were quality controlled and harmonized before analysis to ensure consistency in SNP alignment, allele frequency matching, and reference genome build. These large-scale GWAS datasets provided sufficient statistical power for causal inference and sensitivity analyses.

### 2.3. Selection of IVs

To ensure the validity of the MR analysis, robust IVs were constructed by selecting SNPs associated with each lipid metabolite. SNPs were selected based on a genome-wide significance threshold of *p* < 1 × 10^−5^ [16, 21, 22]. To ensure independence among SNPs and minimize potential bias due to linkage disequilibrium (LD), clumping was performed using a pairwise LD threshold of *R*^2^ < 0.01 within a 1000 kb window [22]. To further reduce the risk of weak instrument bias, we calculated the F-statistic for each SNP. SNPs with F-statistics ≤ 10 were excluded, consistent with conventional thresholds indicating sufficient instrument strength. The proportion of variance explained (*R*^2^) and F-statistics were calculated using the following formulas: *R*^2^ = 2 × β^2^ × EAF × (1 − EAF); F = (N − 2) × *R*^2^/(1 − *R*^2^), where β denotes the SNP effect size on the exposure, EAF is the effect allele frequency, and N refers to the sample size of the metabolite GWAS. Only SNPs meeting both LD independence and instrument strength criteria (*F* > 10) were retained for downstream MR analyses. This rigorous selection process ensured that the relevance assumption of MR was satisfied and that the selected SNPs provided a robust genetic proxy for each exposure.

### 2.4. MR Analysis

MR analyses were conducted to investigate the potential causal effects of 386 circulating lipid metabolites on the risk of ER+ and ER− breast cancer. The inverse variance weighted (IVW) method served as the primary analytical approach and was implemented using the mr function from the TwoSampleMR package (Version 0.6.17) in R (Version 4.4.1) [23]. The IVW method combines SNP-specific Wald ratio estimates using a fixed-effects meta-analysis framework, under the assumption that all genetic instruments are valid and there is no directional horizontal pleiotropy. This method yields an unbiased causal estimate when all IVs satisfy the core MR assumptions. Effect estimates were weighted by the inverse of their variance to optimize statistical efficiency. To account for the potential inflation of type I error due to multiple comparisons across a large number of exposures, *p* values from IVW analyses were corrected using the Benjamini–Hochberg procedure to control the FDR. Associations with an FDR-adjusted *p* < 0.05 were considered statistically significant [16, 24].

### 2.5. Sensitivity and Validation Analyses

To ensure the robustness and reliability of the causal associations identified between lipid metabolites and ER+ breast cancer risk, a series of sensitivity analyses were conducted. Horizontal pleiotropy was evaluated using the MR-Egger regression method, in which a statistically significant deviation of the intercept term from zero (*p* < 0.05) was considered indicative of directional pleiotropy. Heterogeneity among IVs was assessed using Cochran's *Q* statistic under both the IVW and MR-Egger frameworks. A *p* > 0.05 was interpreted as a lack of significant heterogeneity, supporting the homogeneity of SNP-specific causal effects. Moreover, funnel plots were generated to visually assess the presence of directional horizontal pleiotropy. These plots display individual SNP effect estimates against their corresponding standard errors. A symmetric funnel shape centered around the pooled estimate indicates the absence of directional pleiotropy, while asymmetry may suggest potential pleiotropic effects. To assess the influence of individual SNPs on the overall causal estimates, a leave-one-out analysis was performed. In this approach, each SNP was sequentially removed from the instrument set, and the MR analysis was repeated to evaluate the stability of the causal effect estimate. A substantial change in the effect size or direction following the exclusion of a single SNP was interpreted as evidence of that SNP being an influential outlier.

In addition, an external validation analysis was performed to replicate the primary findings using an independent GWAS dataset for ER+ breast cancer (ieu-a-1132). The same IVs and analytical framework were applied. Concordance in the direction and magnitude of effect estimates, as well as statistical significance across both the primary and validation datasets, was used to assess the reproducibility and generalizability of the observed associations.

### 2.6. Reverse MR Analysis

To assess potential reverse causality between ER+ breast cancer and the identified lipid metabolites, reverse MR analyses were performed. In this framework, ER+ breast cancer was considered the exposure, while the three lipid metabolites—myristoleate (14:1n5), tricosanoyl sphingomyelin (d18:1/23:0), and 5α-androstan-3β,17β-diol monosulfate (2)—were treated as individual outcomes. Genetic instruments for ER+ breast cancer were extracted from the publicly available GWAS summary dataset (ieu-a-1127) using the extract_instruments function from the TwoSampleMR R package (Version 0.6.17) [23]. Instrument selection was based on a genome-wide significance threshold (*p* < 5 × 10^−8^), and LD pruning was applied with an *R*^2^ threshold of 0.001 within a 10,000 kb window to ensure independence among variants [21]. To avoid weak instrument bias, only SNPs with F-statistics greater than 10 were retained. The primary causal estimates were derived using the IVW method via the mr function, which assumes all instruments are valid and there is no horizontal pleiotropy. Sensitivity analyses—including heterogeneity testing, MR-Egger regression, and leave-one-out analysis—were conducted in parallel with those used in the forward MR framework to evaluate the robustness and reliability of the findings.

### 2.7. Statistical Analysis

All data analyses were performed using R software (Version 4.4.1). The R packages TwoSampleMR (Version 0.6.17), ieugwasr (Version 1.0.4), VariantAnnotation (Version 1.52.0), gwasglue (Version 0.0.0.9000), circlize (Version 0.4.16), and forestploter (Version 1.1.3) were utilized for various stages of the analysis. Statistical significance was defined as *p* < 0.05.

## 3. Results

### 3.1. Selection and Validation of Reliable Genetic Instruments

To identify suitable IVs for each circulating blood lipid metabolite, SNPs were selected based on a significance threshold of *p* < 1 × 10^−5^. To ensure independence among SNPs, clumping was performed using an *R*^2^ threshold of 0.01 within a 1000 kb window. Detailed information on the selected IVs for each metabolite, including SNP identifiers, effect alleles, and clumping results, is provided in Table S2.

To mitigate potential bias from weak instruments, F-statistics were calculated for all included SNPs. The F-values ranged from 19.507 to 1820.892 (Table S2), with all values exceeding the conventional threshold of 10, indicating that the selected IVs were sufficiently strong and unlikely to introduce weak instrument bias into the MR analysis. This rigorous selection strategy ensured that the IVs met the relevance assumption of MR and provided a robust foundation for subsequent causal inference.

### 3.2. MR Analysis

**Figure 2:**
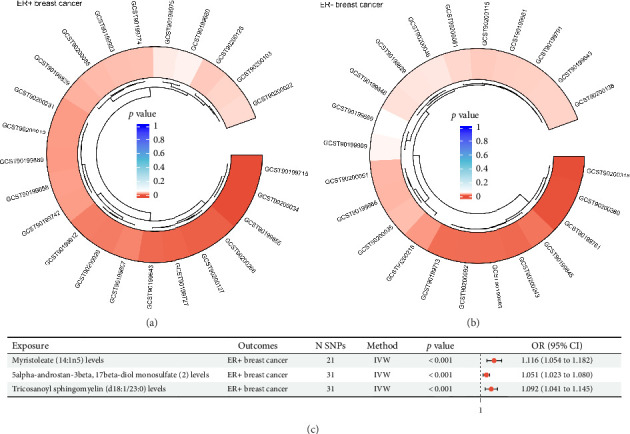
MR analysis of 386 lipid metabolites with ER+ and ER− breast cancer. (a, b) Circos plots illustrating lipid metabolites with *p* < 0.05 from MR analyses using the IVW method, assessing the causal associations between 386 lipid metabolites and ER+ or ER− breast cancer. (c) Forest plot showing the three lipid metabolites significantly associated with ER+ breast cancer after FDR correction (FDR < 0.05). CI, confidence interval; ER, estrogen receptor; FDR, false discovery rate; IVW, inverse variance weighted; MR, Mendelian randomization; SNP, single-nucleotide polymorphism; OR, odds ratio.

To evaluate the potential causal effects of circulating blood lipid metabolites on the risk of ER+ and ER− breast cancer, we performed a two-sample MR analysis. The previously identified robust IVs were utilized, and the IVW method served as the primary analytic approach. Each lipid metabolite was treated as an exposure, with ER+ and ER− breast cancer analyzed separately as outcomes. In the primary MR analysis, 24 lipid metabolites showed nominally significant associations (*p* < 0.05) with the risk of ER+ breast cancer (Table S3, Figure 2(a)), while 23 metabolites were similarly associated with ER− breast cancer (Table S4, Figure 2(b)). To account for multiple comparisons, we applied FDR correction, with FDR < 0.05 considered statistically significant. Following FDR correction, three lipid metabolites remained significantly associated with ER+ breast cancer: myristoleate (14:1n5), tricosanoyl sphingomyelin (d18:1/23:0), and 5α-androstan-3β, 17β-diol monosulfate (2) (all FDR < 0.05; Table S3, Figure 2(c)). These associations suggest potential causal roles of specific lipid metabolic pathways in the development of ER+ breast cancer. In contrast, none of the associations with ER− breast cancer passed the FDR threshold for statistical significance (Table S4), implying that the nominal associations observed prior to correction may not represent robust causal relationships. Collectively, these findings support the hypothesis that lipid metabolism may play a more prominent causal role in the etiology of ER+ breast cancer, underscoring the molecular heterogeneity between breast cancer subtypes.

### 3.3. Sensitivity and Validation Analyses

**Figure 3:**
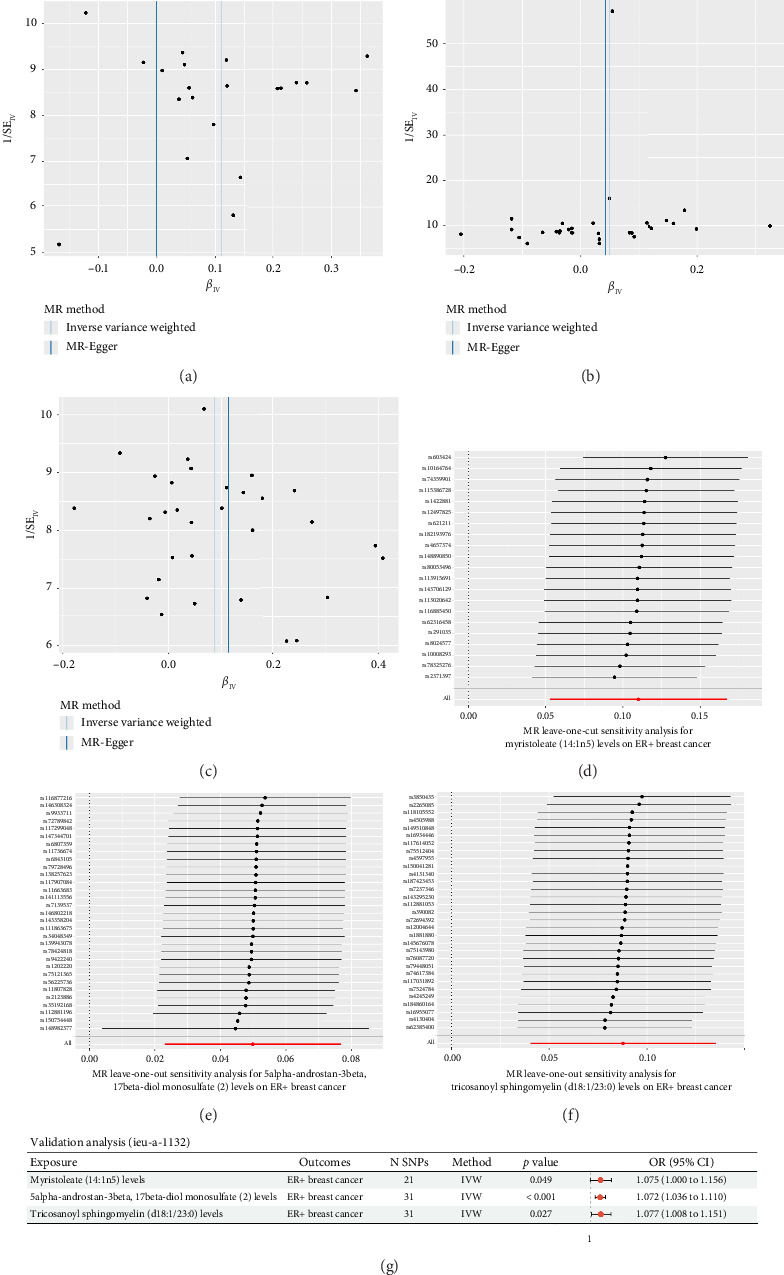
Sensitivity and validation analyses for three ER+ breast cancer-associated lipid metabolites. (a–c) Funnel plots assessing the presence of directional pleiotropy for three lipid metabolites (myristoleate [14:1n5], 5alpha-androstan-3beta, 17beta-diol monosulfate [2], and tricosanoyl sphingomyelin [d18:1/23:0]) in association with ER+ breast cancer using the IVW and MR-Egger methods. (d–f) Leave-one-out sensitivity analysis plots showing the stability of causal estimates for each SNP removed iteratively in the MR analysis of the three metabolites. (g) Forest plots from independent validation analyses confirming the associations of the three metabolites with ER+ breast cancer. All three showed consistent effect estimates with statistical significance using the IVW method. CI, confidence interval; IVW, inverse variance weighted; MR, Mendelian randomization; SE, standard error; SNP, single nucleotide polymorphism; OR, odds ratio.

To assess the robustness and reliability of the MR findings, sensitivity analyses were performed for the three lipid metabolites that showed statistically significant associations with ER+ breast cancer after FDR correction. Specifically, horizontal pleiotropy was evaluated using the MR-Egger intercept test, and heterogeneity was assessed using Cochran's *Q* statistic. For myristoleate (14:1n5), tricosanoyl sphingomyelin (d18:1/23:0), and 5α-androstan-3β, 17β-diol monosulfate (2), both the MR-Egger intercept and Cochran's *Q* tests yielded *p* > 0.05, indicating no evidence of horizontal pleiotropy or heterogeneity (Table 1, Figures 3(a), 3(b), 3(c)). To further validate the stability of the results, a leave-one-out sensitivity analysis was conducted for each metabolite. Sequential exclusion of individual SNPs did not substantially alter the causal estimates, suggesting that the observed associations were not driven by any single IV and that the findings were robust (Figures 3(d), 3(e), 3(f)). In total, 21 SNPs were used as IVs for myristoleate (14:1n5), 31 SNPs for tricosanoyl sphingomyelin (d18:1/23:0), and another distinct set of 31 SNPs for 5α-androstan-3β, 17β-diol monosulfate (2) (Figures 3(d), 3(e), 3(f)).

In addition, a validation MR analysis was performed using an independent GWAS dataset of ER+ breast cancer (ieu-a-1132) to replicate the identified associations. The same three lipid metabolites were used as exposures, and all three associations remained statistically significant (*p* < 0.05, Figure 3(g)). No evidence of heterogeneity or horizontal pleiotropy was observed in the validation analysis (Table S5), further supporting the robustness and reproducibility of the causal relationships between these lipid metabolites and ER+ breast cancer.

### 3.4. Reverse MR Analysis

**Figure 4:**
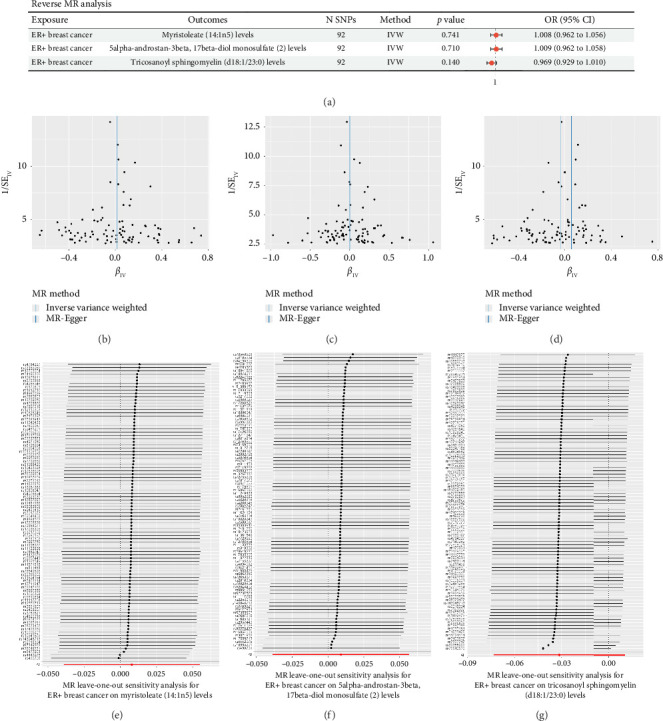
Reverse MR analysis and sensitivity assessments for ER+ breast cancer and three lipid metabolites. (a) Forest plot summarizing the reverse MR analysis evaluating the potential causal effects of ER+ breast cancer on levels of three lipid metabolites: myristoleate (14:1n5), 5alpha-androstan-3beta,17beta-diol monosulfate (2), and tricosanoyl sphingomyelin (d18:1/23:0). (b–d) Funnel plots assessing directional pleiotropy for the three lipid metabolites using the IVW and MR-Egger methods. (e–g) Leave-one-out sensitivity analyses demonstrating the robustness of reverse MR results, with each SNP iteratively removed from the analysis. CI, confidence interval; IVW, inverse variance weighted; MR, Mendelian randomization; SNP, single-nucleotide polymorphism; SE, standard error; OR, odds ratio.

To evaluate the possibility of reverse causality—namely, whether ER+ breast cancer exerts causal effects on the levels of the three identified lipid metabolites—a reverse MR analysis was conducted. A total of 92 SNPs associated with ER+ breast cancer were selected as IVs based on the default clumping criteria. The IVW method was used as the primary approach in the reverse MR analysis. No statistically significant associations were observed between ER+ breast cancer and any of the three lipid metabolites, suggesting no evidence of reverse causality (Figure 4(a)). Tests for horizontal pleiotropy using the MR-Egger intercept and for heterogeneity using Cochran's *Q* statistic both yielded *p* > 0.05, indicating that the IV assumptions were not violated (Table S6). Visual inspection of funnel plots demonstrated symmetric distributions of SNP effects around the IVW estimate, further supporting the absence of horizontal pleiotropy (Figures 4(b), 4(c), 4(d)). Additionally, leave-one-out sensitivity analysis showed that the exclusion of individual SNPs did not materially affect the causal estimates, confirming the stability and robustness of the reverse MR findings (Figures 4(f) and 4(g)). These results collectively suggest that the previously observed associations between lipid metabolites and ER+ breast cancer are unlikely to be driven by reverse causation.

## 4. Discussion

In this two-stage MR study, we systematically evaluated the causal roles of 386 circulating lipid metabolites in the development of ER+ and ER− breast cancer. Our findings provide new evidence supporting a potential etiological role of lipid metabolism in ER+ breast cancer. Specifically, we identified three lipid metabolites—myristoleate (14:1n5), tricosanoyl sphingomyelin (d18:1/23:0), and 5α-androstan-3β,17β-diol monosulfate (2)—that demonstrated significant and reproducible causal associations with ER+ breast cancer after FDR correction. In contrast, no lipid metabolites showed statistically significant causal associations with ER− breast cancer. Reverse MR analysis further supported the directionality of these associations, ruling out reverse causality from ER+ breast cancer to metabolite levels.

Compared with previous MR studies such as that of Lin et al. [16], which adopted a relatively lenient criterion for IV selection (LD threshold of *R*^2^ < 0.1 and a clumping window of 500 kb), our study employed a more stringent filtering strategy, aligned with the methodology proposed by Yin et al. [22]. Specifically, we applied a tighter LD threshold (*R*^2^ < 0.01) and an expanded clumping distance of 1000 kb to ensure greater independence among selected SNPs. This conservative selection framework minimizes the risk of weak instrument bias, reduces potential confounding from correlated variants, and enhances the specificity of causal inference. By improving the validity of the IVs, our approach reinforces the robustness and credibility of the observed associations between lipid metabolites and ER-defined breast cancer subtypes. In contrast to Yin et al. [22], who predominantly utilized the MR-RAPS framework with inherent robustness against horizontal pleiotropy—supplemented by MR-Egger, MR-PRESSO global, and Steiger filtering—our study employed a distinct and complementary analytic strategy. Specifically, we applied Cochran's *Q* statistics derived from both the IVW and MR-Egger models to assess heterogeneity and potential pleiotropy, alongside leave-one-out sensitivity analysis, reverse MR, and replication across independent datasets to reinforce the robustness of our causal inference. In terms of outcomes, whereas Yin et al. conducted a large-scale metabolome-wide screen involving 1099 metabolites and 2099 binary disease endpoints [22], our study adopted a more focused approach by specifically examining 386 well-characterized circulating lipid metabolites. This targeted design allowed for a deeper investigation into lipid metabolic dysregulation and its potential causal role in ER-stratified breast cancer. Through stratified MR analyses, we uncovered subtype-specific associations that offer refined insights into the distinct metabolic etiologies of ER+ and ER− breast cancers. Notably, we identified several novel lipid-related metabolites—myristoleate (14:1n5), tricosanoyl sphingomyelin (d18:1/23:0), and 5α-androstan-3β,17β-diol monosulfate (2)—as potential causal factors, none of which were highlighted in the Yin et al. analysis [22]. These findings not only validate previously suggested links between lipid metabolism and breast cancer risk but also reveal new, biologically plausible, subtype-specific metabolic signatures. Collectively, our study underscores the strength of a focused lipidomic MR framework in uncovering mechanistic insights into hormone receptor–defined breast cancer subtypes.

Our study extends the current understanding of circulating lipid metabolites in the risk of breast cancer by leveraging MR methodology, which reduces biases from confounding and reverse causation. Previous observational studies have linked dysregulated lipid profiles with increased breast cancer risk [25, 26], but such associations may be confounded by lifestyle, comorbidities, or disease-induced metabolic changes. Our MR approach, using genetic proxies for exposure, offers stronger causal inference and highlights specific metabolites likely involved in ER+ breast cancer. Among the identified metabolites, myristoleate (14:1n5) is a monounsaturated fatty acid implicated in membrane structure and signaling. Epidemiological evidence from a prospective cohort study by Sczaniecka et al. [27], using Cox proportional hazards models, demonstrated that increased dietary intake of myristoleic acid was significantly associated with elevated breast cancer risk. Our MR analysis corroborates this finding from a genetic perspective, particularly in the ER+ breast cancer subtype. This association is further supported by recent evidence from Wei et al. [28], who reported a positive correlation between genetically predicted myristoleate levels and breast cancer risk. Despite consistent observational and genetic findings, the underlying biological mechanisms remain to be elucidated through mechanistic and experimental studies. Tricosanoyl sphingomyelin (d18:1/23:0), a very long-chain sphingomyelin species, is a structural constituent of lipid rafts—cholesterol- and sphingolipid-rich microdomains that regulate membrane protein localization and cellular signaling. Notably, Wei et al. reported that levels of tricosanoyl sphingomyelin mediated the causal effect of immune cell traits on ER+ breast cancer but not on ER− tumors [28], aligning with our findings that highlight its potential subtype-specific role. These data suggest that lipid microdomain composition may influence hormone receptor–specific tumor development. 5α-androstan-3β,17β-diol monosulfate (2) is a sulfated metabolite derived from dihydrotestosterone (DHT), representing an inactive, circulating form of androgen [29, 30]. This metabolite has been proposed as a marker of androgenic activity and intracrine steroid metabolism. Given the complex interplay between androgen and ER signaling pathways [31], especially in ER+ breast cancer, its association with increased risk may reflect hormonal crosstalk that contributes to tumor progression. Yue et al. also found a significant association between 5α-androstan-3β,17β-diol monosulfate (2) and increased risk of HER2-positive breast cancer (odds ratio [OR] = 1.07, 95% confidence interval [CI]: 1.03–1.12, *p*=0.0012) [29], although the study did not apply multiple testing correction. In contrast, our study employed stringent statistical thresholds, including FDR correction and independent cohort validation, to ensure the robustness of this association within the ER+ breast cancer context. Nonetheless, further mechanistic research is needed to clarify the functional role of this androgen metabolite in breast cancer pathogenesis.

Importantly, our findings highlight the metabolic heterogeneity between ER+ and ER− breast cancer subtypes. Although both subtypes may share overlapping genetic predispositions and environmental exposures, our MR results reveal a more pronounced and specific causal relationship between circulating lipid metabolites and ER+ breast cancer. This observation aligns with prior studies demonstrating that ER+ tumors exhibit distinct metabolic profiles, particularly in lipid utilization and hormone-responsive pathways [32–35]. The preferential association of lipid dysregulation with ER+ breast cancer may be attributable to the interplay between lipid signaling and ER activity, which could influence tumor initiation and progression. These insights carry meaningful clinical implications—suggesting that lipid metabolism–based biomarkers or interventions may offer greater utility in risk stratification, early detection, and prevention for ER+ breast cancer specifically. Future research should explore the mechanistic basis of this metabolic specificity and assess whether targeted metabolic modulation could serve as a viable strategy in subtype-specific breast cancer prevention and therapy.

Our study has several strengths. First, we leveraged a comprehensive panel of 386 circulating lipid metabolites derived from a large-scale, high-quality GWAS dataset, offering extensive coverage of lipid metabolic pathways. Second, the implementation of a two-sample MR design, combined with rigorous replication in independent GWAS datasets, enhances the credibility and generalizability of our findings. Robustness was further supported through multiple sensitivity analyses, including MR-Egger regression to detect potential directional pleiotropy, Cochran's *Q* statistic to assess heterogeneity, and leave-one-out analyses to examine the influence of individual IVs. Third, to address concerns of potential reverse causality, we conducted reverse-direction MR analyses, which showed no significant evidence of feedback effects from breast cancer to lipid metabolite levels.

Nevertheless, some limitations should be acknowledged. First, our analysis was restricted to individuals of European ancestry, which may limit generalizability to other populations. Second, the MR approach assumes linear and time-invariant effects of exposures, which may overlook potential nonlinear relationships or stage-specific roles of lipid metabolites during breast cancer progression. Third, although we identified significant associations, the biological roles of these metabolites in breast cancer progression remain to be experimentally validated.

## 5. Conclusion

In conclusion, this MR study identified three circulating lipid metabolites—myristoleate (14:1n5), tricosanoyl sphingomyelin (d18:1/23:0), and 5α-androstan-3β,17β-diol monosulfate (2)—as putative causal factors for ER+ breast cancer, with results validated in an independent GWAS dataset. The absence of reverse causality and subtype specificity suggests these metabolites may serve as potential biomarkers or therapeutic targets for ER+ breast cancer.

## Figures and Tables

**Table 1:** Sensitivity analysis of the causal association between metabolites or metabolite ratios and breast cancer risk.

Exposure	Outcomes	Pleiotropy		Heterogeneity			
		MR-Egger		Inverse variance weighted		MR-Egger	
		Intercept	*p*	*Q*	*p*	*Q*	*p*
Myristoleate (14:1n5) levels	ER+ breast cancer (ieu-a-1127)	0.011	0.130	25.469	0.184	22.506	0.260
5alpha-androstan-3beta,17beta-diol monosulfate (2) levels	ER+ breast cancer (ieu-a-1127)	0.002	0.564	33.645	0.295	33.255	0.268
Tricosanoyl sphingomyelin (d18:1/23:0) levels	ER+ breast cancer (ieu-a-1127)	−0.003	0.588	35.352	0.230	34.990	0.205

## Data Availability

The datasets used in this study are publicly available summary datasets and can be found in the cited papers, the GWAS Catalog database (https://www.ebi.ac.uk/gwas/home), and the IEU GWAS database. There are no restrictions on data availability other than those imposed by the corresponding data committee.

## Supporting Information

Table S1: Names of the 386 lipid metabolites and links to download the corresponding GWAS data.

Table S2: *F*-test of IVs for 386 lipid metabolites.

Table S3: MR analysis of 386 circulating lipid metabolites in relation to ER+ breast cancer risk, including FDR-adjusted *p* values.

Table S4: MR analysis of 386 circulating lipid metabolites in relation to ER− breast cancer risk, including FDR-adjusted *p* values.

Table S5: Sensitivity analyses of MR for three validated lipid metabolites and ER+ breast cancer in the replication dataset.

Table S6: Sensitivity analyses of reverse MR between ER+ breast cancer and the three identified lipid metabolites.
